# Associations between the waist-to-height ratio index and migraine: A cross-section study of the NHANES 1999–2004

**DOI:** 10.1371/journal.pone.0312321

**Published:** 2024-10-23

**Authors:** Jing Jin, Yafang Zheng, Tianqi Gao, Xuanyu Lin, Shi Li, Chunyuan Huang

**Affiliations:** 1 Liaoning University of Traditional Chinese Medicine, Shenyang, China; 2 Zhejiang Chinese Medical University, Hangzhou, China; University of Montenegro-Faculty of Medicine, MONTENEGRO

## Abstract

**Background:**

The importance of obesity as a factor that increases the probability of migraine episodes is increasingly acknowledged. Thus, this study aimed to explore the potential correlation between central obesity and migraine, emphasizing the waist-to-height ratio (WHtR) as a key measure in assessing this relationship.

**Methods:**

This cross-sectional analysis included 13,344 individuals who participated in the National Health and Nutrition Examination Survey (NHANES) from 1999–2004. To investigate the association associations between WHtR and migraine, we utilized refined multivariate logistic regression models, smoothing curve fitting methods, subpopulation analysis, and interactive testing.

**Results:**

Of the 13,344 participants, 2,764 (20.72%) had migraines. A significant positive correlation was observed between the WHtR and migraine incidence in both the partially adjusted model (3.08 [95% CI: 1.92–4.94]) and the crude model (1.95 [95% CI: 1.23–3.08]). The participants in the highest quartile of the WHtR had a 13% greater incidence of migraine than those in the lowest quartile [1.13(0.99,1.28)]. The interaction analysis revealed a statistically significant difference (*p*<0.01) in this relationship among the subgroups. Notably, the correlation between WHtR and migraine risk was not significant and negative in patients ≥60 years, indicating that obesity has a mitigating role in preventing migraine in this elderly population.

**Conclusions:**

The incidence of migraine increased concomitantly with increased WHtR. However, central obesity has a protective effect against migraine occurrence in individuals ≥60 years. Thus, our findings underscore the importance of WHtR in migraine prevention and management strategies and highlight its potential as a critical biomarker for mitigating migraine incidence.

## Background

A global burden of disease (GBD) study revealed that migraine is a significant global public health concern and one of the most prevalent and debilitating medical conditions [1]. Fifteen percent of adults in the United States endure migraine attacks each year. This disorder is characterized by recurrent intense headache episodes and significantly impacts a person’s overall quality of life [2]. Therefore, identifying modifiable or preventable factors is critical for minimizing the morbidity associated with migraine. A global surge in the prevalence of obesity, encompassing >200 million individuals categorized as overweight and representing about 30% of the world’s population, has been reported [3]. Despite the complexities of migraine pathogenesis, the obesity-related inflammatory milieu significantly exacerbates migraine symptoms [4]. Consequently, a thorough correlation analysis between obesity and migraine incidence could provide crucial insights into the potential interplay between them.

Traditional obesity metrics, such as body mass index and waist circumference (WC) present certain limitations. The utilization of the Body Mass Index (BMI) is inadequate for distinguishing individuals with excessive adipose tissue and those with a higher muscular composition, and it fails to explain the variations in body fat distribution [5]. Although WC is a central obesity indicator, its precision is significantly impacted by variables such as age and height. Thus, stature and the manifestation of central obesity should be considered in individuals with elevated metabolic risk. Given this context, the WHtR can be a potentially advantageous alternative metric that comprehensively assesses obesity-related health risks [6]. A prospective investigation and meta-analyses have shown that the WHtR is either equivalent to marginally better than WC and surpasses BMI in predicting increased cardiometabolic risk [7]. The pathogenesis of migraine is complex, with obesity status considered a significant and critical risk factor [8]. Although the WHtR is well known as an effective tool for predicting the risk of several cardiovascular diseases, to date, there is a paucity of research on the possible association between WHtR and migraine. If this potential association could be scientifically validated, it could provide a novel and powerful tool for healthcare professional teams in dealing with migraine attack management and care strategies. Therefore, the present study aimed to investigate whether there is a significant association between WHtR and migraine headaches through a rigorous cross-sectional analysis based on the rich data resources of the NHANES from 1999–2004, with the aim of providing new perspectives and rationales for the prevention and treatment of migraine headache in the future.

## Materials and methods

### Study population

Conducted by the Centers for Disease Control and Prevention (CDC), the NHANES is a unique program that uses interviews, physical examinations, and laboratory evaluations to assess the health and nutritional status of American participants [9]. Thus, a stratified multistage probability sampling method was adopted to create a representative sample reflecting the nation’s diverse demographic traits. This methodology guarantees the reliability and validity of the results when they are extrapolated to other American populations [10]. All study participants underwent rigorous home interviews, encompassing comprehensive physical examinations and laboratory assessments. Before the study commenced, these procedures were assessed and approved by the Research Ethics Review Committee of the National Center for Health Statistics (NCHS) in compliance with the strictest ethical norms. Furthermore, all participants provided written informed consent for ethical soundness [11]. A comprehensive synopsis of the NHANES study is available on the official website at https://www.cdc.gov/nchs/nhanes/, providing comprehensive details regarding the program’s range and research approach.

**Fig 1:**
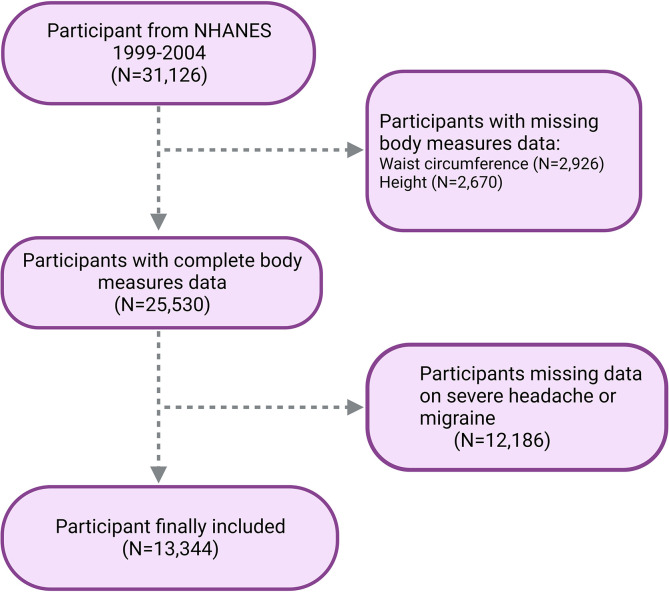
Flow chart of participants selection. NHANES, National Health and Nutrition Examination Survey.

Thus, we used the NHANES dataset from 1999–2004 for analysis. Our study specifically included participants whose comprehensive data concerning migraine episodes and WHtR were available. Initially, we recruited 31,126 individuals. However, we excluded those with missing or incomplete data regarding WC (n = 2,926), height (n = 2,670), and migraine (n = 12,186) to maintain accuracy. Consequently, our final cohort comprised 13,344 participants (Fig 1).

### Definition of WHtR

The WHtR is a popular and novel assessment indicator. It is a reliable marker for evaluating obesity levels, with a higher score indicating greater obesity rates. More specifically, the WHtR is obtained by dividing an individual’s WC in centimeters by their height [7]. Anthropometric assessments, administered by qualified medical staff in mobile examination facilities, were recorded by dedicated professionals for data integrity. A digital scale is employed for accurate weight measurements. Before the weighing procedure, the participants were dressed in clothing suitable for the examination, and then stood without shoes in the middle of the calibrated scale, keeping their arms at their sides and a forward gaze. This standardized protocol guarantees reliable weight measurements for subsequent analysis [12]. For the anthropometric assessments, a tape measure was used to determine WC precisely. To ensure accuracy, the tape was positioned precisely at the intersection of the midline extending from the axillary region and the horizontal line superior to the right kneecap’s outermost border [13]. We also designated WHtR as the primary exposure variable under investigation.

### Migraine definition

A comprehensive questionnaire was utilized to evaluate migraine-associated symptoms, emphasizing various manifestations and attributes of pain. The participants were assumed to have experienced migraine if their response to this query, “During the past three months, did you have severe headaches or migraines?” was affirmative. Thus, migraine was designated as a dependent variable, representing the primary focus of our analysis. Although migraine is not a formal diagnosis following the International Classification of Headache Disorders (ICHD) criteria, the findings of the American Migraine Prevalence and Prevention Study indicate that a significant proportion of individuals reporting severe headaches fulfill the ICHD-II criteria for migraine or probable migraine [14]. This classification has also been previously used in multiple migraine-related analyses, thereby providing a unified front for assessing migraine data [15,16].

### Covariates

We incorporated several covariates that might be effective in revealing the association between WHtR and migraine. These covariates included demographic factors such as age, sex, race, education level, hypertension, smoking, diabetes, the poverty-to-income ratio (PIR), and alcohol consumption in the past 12 months.

### Statistical analysis

The statistical procedures were carried out in strict adherence to the methodological principles and recommendations outlined by the CDC. We evaluated the participants’ distinctive features by segmenting them into WHtR-based quartiles. Furthermore, we applied statistical tests, such as chi-square or t-test, to examine the variations in characteristics across the quartiles. The participants’ demographics were analyzed across WHtR quartiles using the chi-square test. To investigate the linear associations between WHtR and migraine, we utilized multivariate linear regression and logistic regression analyses in three distinct models. Model 1 did not have covariate adjustments. Model 2 involved adjustments for age, sex, and race, while Model 3 was adjusted for age, sex, race, education level, hypertension, PIR, smoking, and alcohol consumption. Following the WHtR score’s categorization from a continuous variable into quartiles, a trend analysis was implemented to scrutinize the potential linear trend between the WHtR and migraine. A sub-stratified analysis was subsequently conducted to explore the potential relationship between WHtR and migraine, considering demographic and clinical variables such as age, sex, race, education level, hypertension, PIR, and smoking and alcohol consumption status. Moreover, interaction tests were conducted to determine the consistency of the observed relationships across various subgroups. Moreover, we employed smooth curve fitting techniques to explore the possible nonlinear correlation between WHtR and migraine [17]. All the statistical analyses were performed via the R package (version 4.2) or EmpowerStats software (version 5.0). Statistical significance was established at a two-tailed p-value <0.05.

## Results

### Baseline characteristics

The current study incorporated NHANES data from 1999–2004. In accordance with the exclusion criteria, 13,344 participants aged ≥20 years were included in the analysis. Table 1 displays the participants’ baseline demographic and clinical features, which were grouped into quartiles according to their WHtR. The mean age of the participants was 49.17 ±18.81 years. Among them, 7,003 individuals (52.48%) were females, and 6,665 (50.31%) were from the non-Hispanic White ethnic group. The prevalence of migraine was 2,764 participants, accounting for 20.72% of the total sample size. Notably, this prevalence tended to increase as the WHtR quartiles increased. The average WHtR across all participants was 0.58 ±0.09. Specifically, the WHtR values for the distinct quartiles were as follows: quartile 1: 0.35–0.52; quartile 2: 0.52–0.58; quartile 3: 0.58–0.6; and quartile 4: 0.64–1.01. Compared with individuals in the lowest WHtR quartile, those in the lowest WHtR quartile presented a significantly greater tendency to belong to the adult population aged >60 years, especially among non-Hispanic White women. Furthermore, they tended toward lower educational attainment, reduced physical activity levels, and a greater risk of developing diabetes and hypertension. Furthermore, a higher WHtR was consistently associated with corresponding increases in BMI, WC, and body height as well as weight. However, the participants presented lower PIRs despite these elevations, as shown in Table 1.

**Table 1:** Basic characteristics of participants by waist-to-height ratio quartile.

Characteristics	Waist-to-height ratio quartile				*P*-value
	Q1 (<0.518) N = 3336	Q2 (0.519–0.577) N = 3336	Q3 (0.578–0.639) N = 3336	Q4 (>0.640) N = 3336	
Age (years)	40.87 ± 17.26	49.13 ± 18.39	53.37 ± 18.57	53.31 ± 18.19	<0.001
Sex, (%)					<0.001
Male	49.73	54.44	50.69	35.22	
Female	50.27	45.56	49.31	64.78	
Race/ethnicity, (%)					<0.001
Non-Hispanic White	54.08	50.15	49.28	46.28	
Non-Hispanic Black	21.85	17.51	16.55	20.47	
Mexican American	15.41	23.32	26.23	26.35	
Other race/multiracial	8.66	9.02	7.94	6.89	
Education level, (%)					<0.001
Less than high school	23.66	30.68	35.64	38.55	
High school	22.85	23.80	23.79	24.60	
More than high school	53.48	45.52	40.56	36.84	
Smoking, (%)					0.015
Ever	47.81	48.68	50.83	47.10	
Never	52.19	51.32	49.17	52.90	
Hypertension, (%)					<0.001
Yes	14.73	25.38	36.91	47.46	
No	85.27	74.62	63.09	52.54	
Diabetes,(%)					<0.001
Yes	2.73	7.92	12.26	20.71	
No	97.27	92.08	87.74	79.29	
Migraines, (%)					0.006
Yes	20.86	19.40	19.94	22.67	
No	79.14	80.60	80.06	77.33	
PIR	2.73 ± 1.60	2.69 ± 1.56	2.58 ± 1.51	2.36 ± 1.49	<0.001
BMI (kg/m^2^)	22.24 ± 2.48	26.06 ± 2.46	29.10 ± 2.93	35.73 ± 5.80	<0.001
Waist circumference (cm)	80.21 ± 6.93	92.31 ± 6.02	100.96 ± 6.62	115.27 ± 11.34	<0.001
Weight (kg)	64.39 ± 11.54	74.43 ± 12.52	81.48 ± 14.33	96.93 ± 21.05	<0.001
Height(cm)	169.61 ± 9.97	168.45 ± 9.74	166.74 ± 9.97	164.15 ± 9.91	<0.001
Exercise over the past 30 days, (%)					<0.001
Yes	51.00	47.87	42.70	37.48	
No	49.00	52.13	57.30	62.52	
Average alcohol consumption past 12 months	2.74 ± 3.86	2.61 ± 2.82	2.65 ± 4.32	2.38 ± 3.38	0.015

Mean ± SD for continuous variables: The P value was calculated by the weighted linear regression model.

(%) for categorical variables: The P value was calculated by the weighted chi-square test.

Abbreviation: Q, quartile; PIR, Ratio of family income to poverty.

### Association between WHtR and migraines

**Fig 2:**
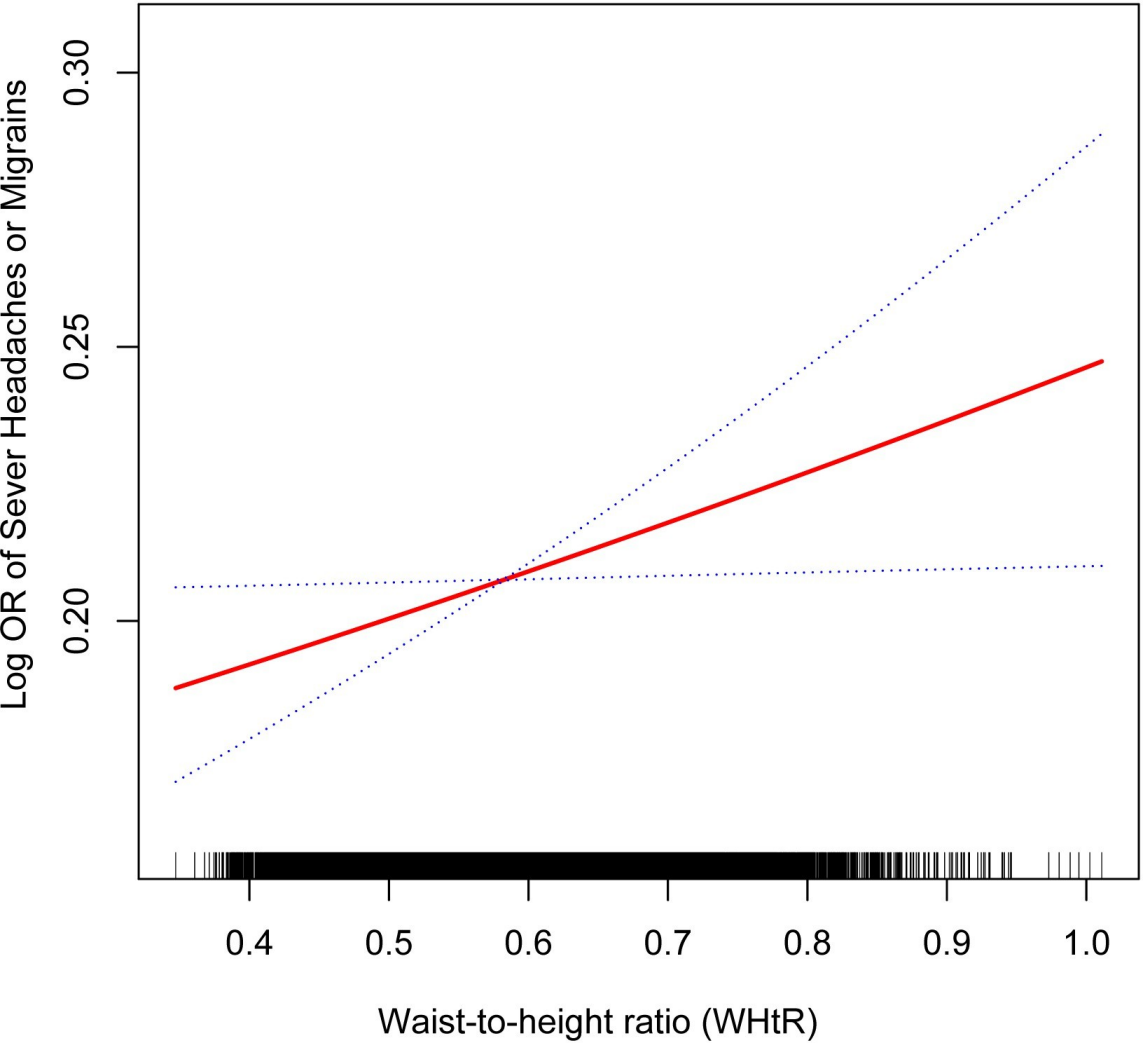
Non-linear positive correlation between WHtR and migraine prevalence. The solid red line indicates a smooth curve fit between the variables. The 95% confidence intervals for the fitted results are indicated by the blue bars.

Table 2 shows the relationship between the WHtR and migraine occurrence. Both the unadjusted [1.95 (1.23, 3.08)] and the partially corrected [3.08 (1.92, 4.94)] models revealed a notable and positive correlation between WHtR and migraine incidence. After adjustments, the aforementioned positive correlation remained statistically significant with an odds ratio of [1.70 (1.04, 2.78)]. Specifically, for every incremental unit in WHtR, the probability of migraine incidence increased by 70%. Following the inclusion of the WHtR quartiles, the positive correlation between WHtR and migraine incidence did not change. Particularly, participants in the highest WHtR quartile presented a 13% greater migraine incidence in comparison to those in the lowest quartile, with an odds ratio of [1.13 (0.99, 1.28)]. Furthermore, the smoothed curve fitting analysis results offered additional verification of the non-linear positive correlation between WHtR and migraine occurrence (Fig 2).

**Table 2:** The associations between waist-to-height ratio and migraines.

Exposure	Model 1 [OR (95% CI)]	Model 2 [OR (95% CI)]	Model 3 [OR (95% CI)]
Waist-to-height ratio (continuous)	1.95 (1.23, 3.08)	3.08 (1.92, 4.94)	1.70 (1.04, 2.78)
Waist-to-height ratio (quartile)			
Quartile 1	reference	reference	reference
Quartile 2	0.91 (0.81, 1.03)	1.11 (0.98, 1.26)	1.09 (0.96, 1.23)
Quartile 3	0.94 (0.84, 1.06)	1.23 (1.08, 1.39)	1.14 (1.01, 1.30)
Quartile 4	1.11 (0.99, 1.25)	1.30 (1.15, 1.47)	1.13 (0.99, 1.28)
P for trend	0.0444	<0.0001	0.0647

Model 1: No covariates were adjusted. Model 2: Age, gender, and race were adjusted. Model 3: Age, gender, race, education level, hypertension, PIR, smoking, and alcohol drinking, were adjusted.

Abbreviation: PIR, Ratio of family income to poverty.

### Subgroup analyses

**Fig 3:**
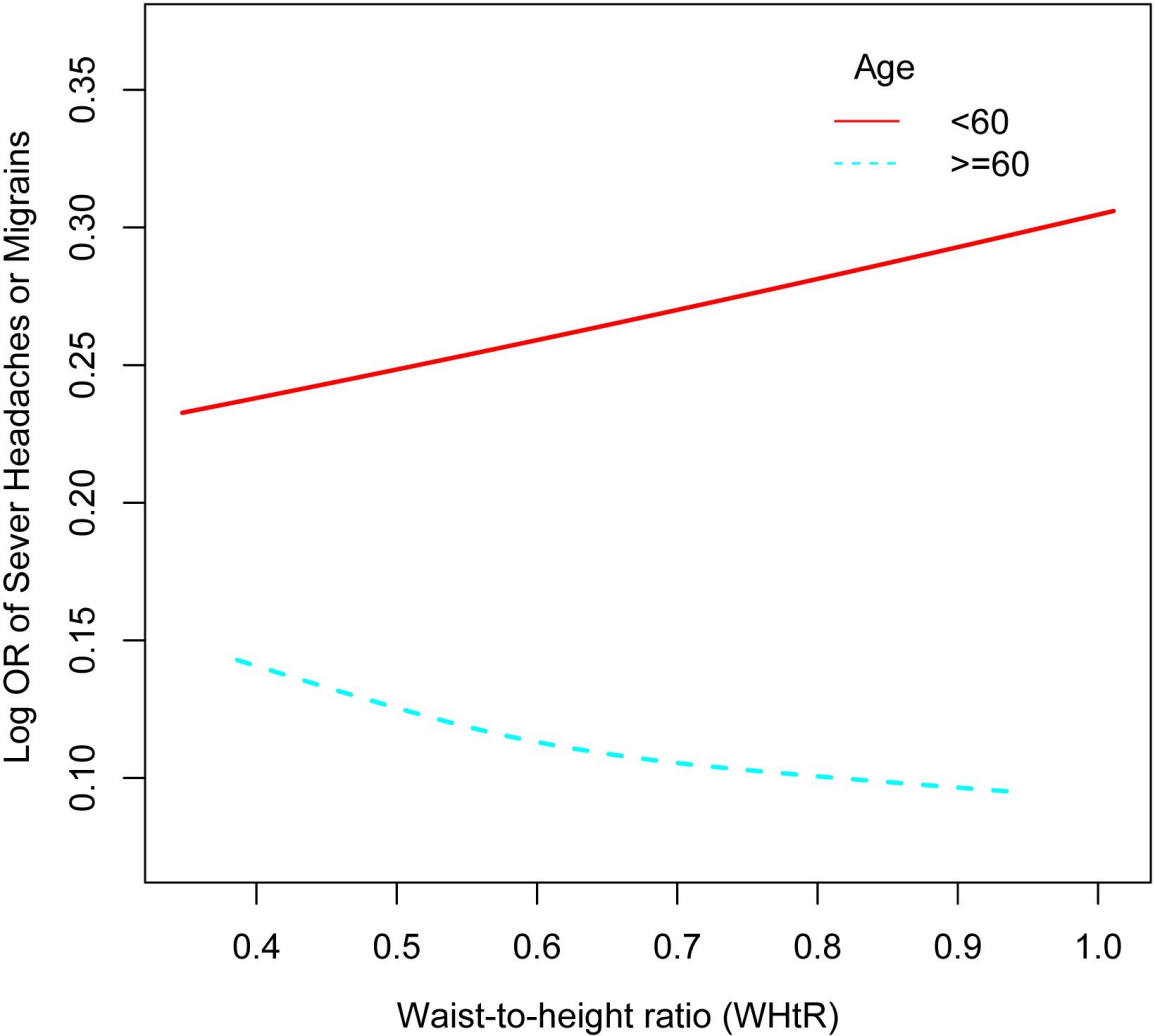
A smooth curve fitting for the age portion of the subgroup analysis showed that WHtR younger than 60 years was positively associated with migraine prevalence and WHtR older than 60 years was negatively associated with migraine prevalence.

We performed subgroup analyses and interaction tests, segmented by gender, race, education, hypertension, PIR, alcohol consumption, and smoking to assess the possible correlation between WHtR and migraine and uncover potential disparities among various population subgroups. The analysis outcomes are presented in Table 3. Our results indicated a statistically significant difference in the association between WHtR and migraine occurrence among different age groups (p-value for interaction <0.01). Among individuals <60 years of age, a unit-wise WHtR increase was linked to an 82% increased likelihood of migraine. In contrast, the previous correlation between WHtR and migraine had a nonsignificant negative association, with a 68% decrease in migraine incidence for each unit increase in WHtR in participants >60 years (Fig 3). Moreover, obesity may have a protective effect against migraines in individuals ≥60 years [18].

**Table 3:** Subgroup analysis of the association between waist-to-height ratio and migraines.

Subgroup	Migraines [OR (95%CI)]	P for interaction
Sex		0.6299
Male	2.04 (0.82, 5.10)	
Female	1.56 (0.87, 2.82)	
Age		0.0087
< 60 years	1.82 (1.06, 3.11)	
≥ 60 years	0.32 (0.10, 1.06)	
Race/ethnicity		0.0452
Non-Hispanic White	2.31 (1.10, 4.84) 0.0273	
Non-Hispanic Black	0.90 (0.33, 2.44) 0.8300	
Mexican American	0.87 (0.30, 2.53) 0.7990	
Other race/multiracial	10.26 (1.88, 56.09) 0.0072	
Education level, n (%)		0.1947
Less than high school	2.03 (0.86, 4.79) 0.1070	
High school	0.81 (0.30, 2.16) 0.6774	
More than high school	2.47 (1.15, 5.30) 0.0199	
PIR		0.6789
<1.3	2.40 (1.01, 5.68) 0.0468	
1.3–3.5	1.45 (0.70, 3.02) 0.3208	
≥1.3	1.68 (0.60, 4.74) 0.3233	
Hypertension, (%)		0.6393
Yes	1.30 (0.55, 3.11) 0.5488	
No	1.68 (0.92, 3.07) 0.0917	
Smoking,(%)		
Ever	1.58 (0.80, 3.13) 0.1849	0.8794
Never	1.71 (0.84, 3.46) 0.1369	

Age, gender, race, education level, hypertension, PIR, alcohol drinking, and smoking were adjusted.

Abbreviation: PIR, Ratio of family income to poverty.

## Discussion

In this extensive cross-sectional study, we utilized 13,344 participants and observed a direct correlation between WHtR and migraine. Furthermore, significant age-related dependency was evident, indicating a complex interplay between WHtR, age, and migraine occurrence. This discovery suggests that higher WHtR values could significantly lead to increased migraine incidence, especially in adults <60 years of age. These observational studies confirmed that an elevated WHtR was an independent and significant risk trigger for migraine occurrence. This important finding further highlights the key role of the WHtR indicator in planning migraine prevention strategies as well as in daily health management.

Our study marks the initial investigation into the relationship between WHtR and migraine, thereby progressing the sphere of inquiry in this area. Many studies have extensively explored the association between WHtR and cardiovascular disease (CVD), highlighting its potential significance in cardiovascular health. In a comprehensive meta-analysis encompassing >300,000 adults, Ashwell et al. established that the WHtR is an exceptional indicator of cardiometabolic risk [7]. Remarkably, the WHtR excelled beyond BMI and WC in precisely recognizing persons at increased risk of diabetes mellitus, hypertension, dyslipidemia, and coronary heart disease, confirming its importance in clinical evaluations [19,20]. According to Romero-Saldaña et al., the WHtR has emerged as the superior indicator for predicting metabolic syndrome (MetS), with a key threshold of 0.54. A NHANES cross-sectional analysis revealed that among U.S. adults with a normal waist circumference but an elevated WHtR, 25.5% were significantly susceptible to diabetes mellitus (OR = 2.06, CI [1.66, 2.55]), hypertension (OR = 1.75, CI [1.58, 1.93]), and CVD (OR = 1.32, CI [1.11, 1.57]) [21]. Furthermore, we revealed a nonlinear positive correlation between WHtR and migraine, aligning with previous research that underscored the adverse impacts of WHtR on cardiovascular health. This finding strengthens the notion that the WHtR can be a crucial indicator for assessing migraine risk and maintaining cardiovascular health. Accumulating research data firmly establishes that obesity is a significant contributing factor to migraine headaches. The WHtR has emerged as a superior indicator for predicting cardiovascular risk, surpassing conventional obesity measures such as BMI or WC when utilized independently. This measurement provides a precise representation of the adverse impacts of central obesity on cardiovascular well-being [22]. Moreover, the WHtR measure is less expensive, easier to use, and has strong academic applicability [23]. An extensive Chinese epidemiological investigation revealed significant variations in the correlation between BMI and migraine occurrence. This dataset underscored the possible impact of BMI fluctuations on the onset and reappearance of migraine attacks [24]. In a comprehensive meta-analysis encompassing 15 observational studies, Ornello et al. revealed that for obese individuals, with every increase of one BMI unit, there was a significant 14% rise in migraine occurrence, along with a considerable 75% surge in the probability of chronic migraine, compared to people with normal weight [25]. Two crucial studies involving NHANES participants have suggested an association between obesity and the frequency of migraine occurrence. Ford et al. evaluated a cohort of 7,601 individuals. Their findings indicated that participants categorized as underweight (BMI<18.5) or obese (BMI≥30) displayed a significantly increased likelihood of enduring severe headaches or migraines, in contrast to those with healthy body weight [26]. In addition, studies have shown that both total body obesity (TBO) and abdominal obesity (AO) are associated with a higher incidence of migraine. These two types of obesity indicators include only BMI and WC and do not consider height or central obesity [27]. Despite a potential link between traditional obesity indicators and migraine, the phenomenon known as the “obesity paradox” continues to persist [19]. Several studies demonstrating the occurrence of the obesity paradox have relied primarily on BMI as the sole obesity assessment measure. However, the relative reduction in central adiposity and lean body mass might be more significant than BMI alone in assessing obesity-related health risks in older adults [28]. The WHtR represents a practical and age-sensitive metric that incorporates height and central adiposity. Thus, as a central obesity measure, the WHtR might offer a more accurate evaluation of migraine-associated risk factors, such as MetS, diabetes, and hypertension [7,29,30].

Numerous investigations have unequivocally demonstrated that diminishing abdominal obesity effectively enhances lipid profiles, reduces hypertension, optimizes glycemic management, and significantly lessens cardiovascular episodes [31,32]. WHtR has become a more precise indicator of obesity, and our study effectively captures the complex link between obesity and migraine, with a 70% increase in migraine incidence for each unit increase in WHtR. Focusing on WHtR values in the clinical setting can help prevent the onset and recurrence of migraine. Existing studies have revealed a possible vicious cycle mechanism between migraine and obesity [33]. Specifically, after a migraine attack, individuals may tend to gain weight due to a variety of factors (e.g., pain-induced activity reduction, medication side effects affecting metabolism, etc.), resulting in obesity [34]. This transition not only exacerbates the physiological burden on the patient but also influences the course of migraine. Obesity, as a systemic metabolic abnormality, is accompanied by pathophysiological changes such as inflammatory responses, hormonal imbalances, and vascular dysfunction, which have been shown to directly or indirectly increase the severity of migraine-related symptoms [8]. For example, obesity may exacerbate neurovascular dysregulation and promote increased sensitivity to migraine triggers, leading to more frequent and uncontrollable migraine attacks [35]. Moreover, migraine-induced obesity may also contribute to the progression of migraine from an episodic, acute state to a chronic state. Chronic migraine not only make patients suffer from headaches for a long time but also significantly reduce their quality of life, increase the consumption of healthcare resources, and may be accompanied by psychological disorders, such as anxiety and depression, which create more complex health challenges. Therefore, an appropriate WHtR is essential for cardiovascular health [36]. This includes maintenance through weight loss diets, ketogenic diets, low-calorie diets, moderate exercise, and weight control [37], which in turn reduce the occurrence and recurrence of migraine [36,38].

Multiple physiological mechanisms may explain the positive association between WHtR and migraine. First, an increased WHtR indicates dysfunctional adipose tissue, marked by a pro-inflammatory condition resulting from increased circulating cytokine concentrations. This inflammation-prone environment likely acts as a catalyst for migraine attacks, further emphasizing the importance of the WHtR as a key indicator for assessing migraine risk [39]. Moreover, p molecules within the adipose tissue help in both the amplification of adipose deposition and the initiation of obesity-induced inflammatory sequences. These proinflammatory states might be interconnected with neurovascular inflammation in migraine sufferers [40]. Third, the increased calcitonin gene-related peptide (CGRP) levels in obese individuals suggest its potential involvement as a significant postsynaptic modulator of trigeminal vascular inflammation in migraine. Moreover, this neuropeptide, a central mediator in the pathological process of chronic migraine, is a highly promising target in the therapeutic strategy of CGRP inhibitors, which, by targeting this neuropeptide, opens new avenues for the relief of chronic migraine symptoms [41]. Finally, adipose tissue secretes a variety of hormones that have a profound effect on the function of the hypothalamus, a key central regulator that is not only responsible for the fine-tuning of body weight and food intake but also involved in triggering migraine attacks, underscoring its central position in the neuroendocrine regulatory network [42]. Thus, obesity-induced alterations in hypothalamic function may alter the incidence of migraines. Hence, weight control is an effective strategy to reduce migraine occurrence; however, psychological and behavioral factors should be considered for preventing migraine.

Moreover, our findings revealed a non-substantial inverse association between WHtR and migraine among individuals aged≥60 years, indicating a lack of significant correlation in this particular cohort. A new study confirms our findings, providing insight into the complex relationship between BMI and WC on mortality in an older age group >80 years, and revealing an inverse correlation between BMI and WC [43]. Additionally, the benefit of decreased all-cause mortality associated with obesity in older adults may be due to protective energy reserves, adipokine concentrations, endotoxin-lipoprotein interactions, or adipotoxin segregation [44].

Our study’s strength lies in the utilization of NHANES data, which were collected via a stratified, multistage probability sampling approach to yield an extensive sample size. This approach ensures the study’s credibility, generalizability, and reliability. Furthermore, subgroup analyses were performed among various populations to obtain more comprehensive insight into the correlation between WHtR and migraine. Nonetheless, our study had a few limitations. Owing to our cross-sectional design, we were unable to conclusively prove a causal relationship between WHtR and migraine. Furthermore, the inclusion criteria in the migraine analysis were based solely on self-reported histories of migraine attacks, and the specific staging phenotype of migraine remained an unknown variable. We could also not explore the potential correlations between distinct migraine subtypes and WHtR, thereby limiting our comprehension of their intricate relationships. Despite several unaccounted confounders, the robust correlation between WHtR and migraine status remained significant without any subsequent alterations.

## Conclusion

In conclusion, our comprehensive analysis revealed a nonlinear, positive association between WHtR and migraine. Central obesity also had a protective effect on deterring migraine episodes among adults >60 years of age. This finding provides evidence for the intricate relationship between body composition and migraine pathogenesis, especially among older adults. Thus, this discovery can significantly contribute novel perspectives toward customized migraine management strategies. However, it is imperative to conduct additional high-quality prospective studies to rigorously validate and substantiate our results.
